# Supplementary figures and images for: Recent population expansion of longtail tuna Thunnus tonggol (Bleeker, 1851) inferred from the mitochondrial DNA markers
Source: PeerJ. 2020 Aug 6;8:e9679. doi: 10.7717/peerj.9679 (PMC7415224; doi:10.7717/peerj.9679)

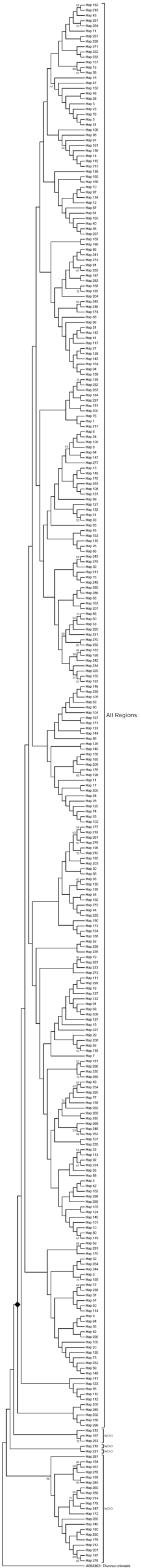

Supplement: Supplemental Information 1 — Hap001-042 (SCS-1), 043-085 (SOM), 086-104 (CS), 108-152 (SCS-2), 153-166 (ECIO), 168-296 (WCIO), 297-300 (TW). [file peerj-08-9679-s001.pdf]
